# Pharmacokinetics of TKM-130803 in Sierra Leonean patients with Ebola virus disease:  plasma concentrations exceed target levels, with drug accumulation in the most severe patients

**DOI:** 10.1016/j.ebiom.2019.102601

**Published:** 2020-01-14

**Authors:** Janet T. Scott, Raman Sharma, Luke W. Meredith, Jake Dunning, Catrin E. Moore, Foday Sahr, Steve Ward, Ian Goodfellow, Peter Horby, RAPIDE-TKM trial team

**Affiliations:** a MRC-University of Glasgow Centre for Virus Research, G61 1QH, UK; b NIHR Health Protection Research Unit in Emerging and Zoonotic Infections, University of Liverpool, Liverpool, UK; c Liverpool School of Tropical Medicine, Liverpool, UK; d Department of Pathology, Division of Virology, University of Cambridge, Cambridge CB2 1QP, UK; e Department of Public Health, University of Makeni, Makeni, Sierra Leone; f National Infection Service, Public Health England, London, UK; g Centre for Tropical Medicine and Global Health, University of Oxford, Oxford, UK; h Republic Sierra Leone Armed Forces, Military Hosp 34, Freetown, Sierra Leone

**Keywords:** TKM, Tekmira, Ebola, Pharmacokinetics

## Abstract

**Background:**

TKM-130803 is a specific anti-EBOV therapeutic comprised of two small interfering RNAs (siRNA) siLpol-2 and siVP35-2. The pharmacokinetics (PK) of these siRNAs was defined in Ebola virus disease (EVD) patients, with reference to efficacy (ET) and toxicology thresholds (TT). The relationship between PK and patient survival was explored.

**Methods:**

Pharmacokinetic (PK) and pharmacodynamic (PD) data were available for seven participants with EVD in Sierra Leone who received 0·3 mg/kg of TKM-130803 by intravenous infusion over 2 h daily for up to 7 days. Plasma concentration of siRNA was compared to survival at 14 days. PK data were fitted to two-compartment models then Monte Carlo simulated PK profiles were compared to ET (Cmax 0·04–0·57 ng/mL and mean concentration 1·43 ng/mL), and TT (3000 ng/mL).

**Findings:**

Viral loads (VL) were not significantly different at treatment onset or during treatment (*p* = 0·1) in subjects who survived or died. siRNA was in quantitative excess of virus genomes throughout treatment, but the 95% percentile exceeded TT. The maximum AUC for which the 95% percentile remained under TT was a continuous infusion of 0·15 mg/kg/day. Plasma concentration of both siRNAs were higher in subjects who died compared to subjects who survived (*p*<0·025 both siRNAs).

**Interpretation:**

TKM-130803 was circulating in molar excess of circulating virus; a level considered needed for efficacy. Given extremely high viral loads it seems likely that the patients died because they were physiologically beyond the point of no return. Subjects who died exhibited some indication of impaired drug clearance, justifying caution in dosing strategies for such patients. This analysis has given a useful insight into the pharmacokinetics of the siRNA in the disease state and illustrates the value of designing PKPD studies into future clinical trials in epidemic situations.

**Funding:**

This work was supported by the Wellcome Trust of Great Britain (grant number 106491/Z/14/Z and 097997/Z/11/A) and by the EU FP7 project PREPARE (602525). The PHE laboratory was funded by the UK Department for International Development. The funders had no role in trial design, data collection or analysis. The views expressed are those of the authors and not necessarily those of Public Health England, the Department of Health, or the EU.

**Trial registration:**

Pan African Clinical Trials Registry PACTR201501000997429.

**Research in context**

**Evidence before this study**

Tekmira (TKM-130803) is an anti-EBOV therapeutic comprised of two small interfering RNAs (siRNA) which inhibit viral replication. Tekmira had successfully protected nonhuman primates from EVD and had undergone safety studies in healthy humans. A trial in EVD subjects in Sierra Leone in 2015 was discontinued, because there was a low probability of overall therapeutic benefit. PK studies are invaluable in considering whether alternative doses or delivery regimens might have been more effective or less toxic. Drug concentrations overtime can be markedly different in patients compared to healthy volunteers, so it is important to carry out these studies in patients. PK studies are extremely challenging during an outbreak, so PK studies were not included in other clinical trials.

**Sources**

This study was informed by previously published literature, sourced by searching online databases using the following key words: Tekmira, TKM, siRNA, Ebola, EBOV, EVD. It was also informed by the investigator's brochure provided by the manufacturer, and WHO recommendations on the characterisation and prioritisation of drugs for consideration or use in patients infected with Ebola.

**Added value of this study**

This study proved that collecting sufficient samples to develop a PK mathematical model was possible in the context of an Ebola outbreak. Subjects had extremely high levels of circulating Ebola virus, over twice that reported in the Zmapp study. The concentration of drug in subjects who died was higher than in subjects who survived, probably because their organs were failing and less able to clear the drug from their body. There were more copies of siRNA in circulation than there were viruses, however the mathematical model indicated that higher dose of drug, even given over a longer period would have unacceptably increased the risk of adverse events.

**Implications of all the available evidence**

**Policy:** PK studies are possible during an Ebola outbreak, yielding useful results with relatively few subjects.

**Practice:** A cautious approach to dosing in advanced EVD patients is warranted, and a reduced drug clearance should be assumed in such patients.

**Future research:** Future clinical trials should aim to recruit subjects with a range of viral loads and consider a stratified approach to analysis. siRNA type therapeutics remain unproven, but promising agents for treating EVD and warrant further study.

**Relevance to human health:**Integrating PK studies into clinical trials for high consequence pathogens should facilitate effective and dose optimised therapeutics.

Alt-text: Unlabelled box

## 1 Introduction

**Fig. 1:**
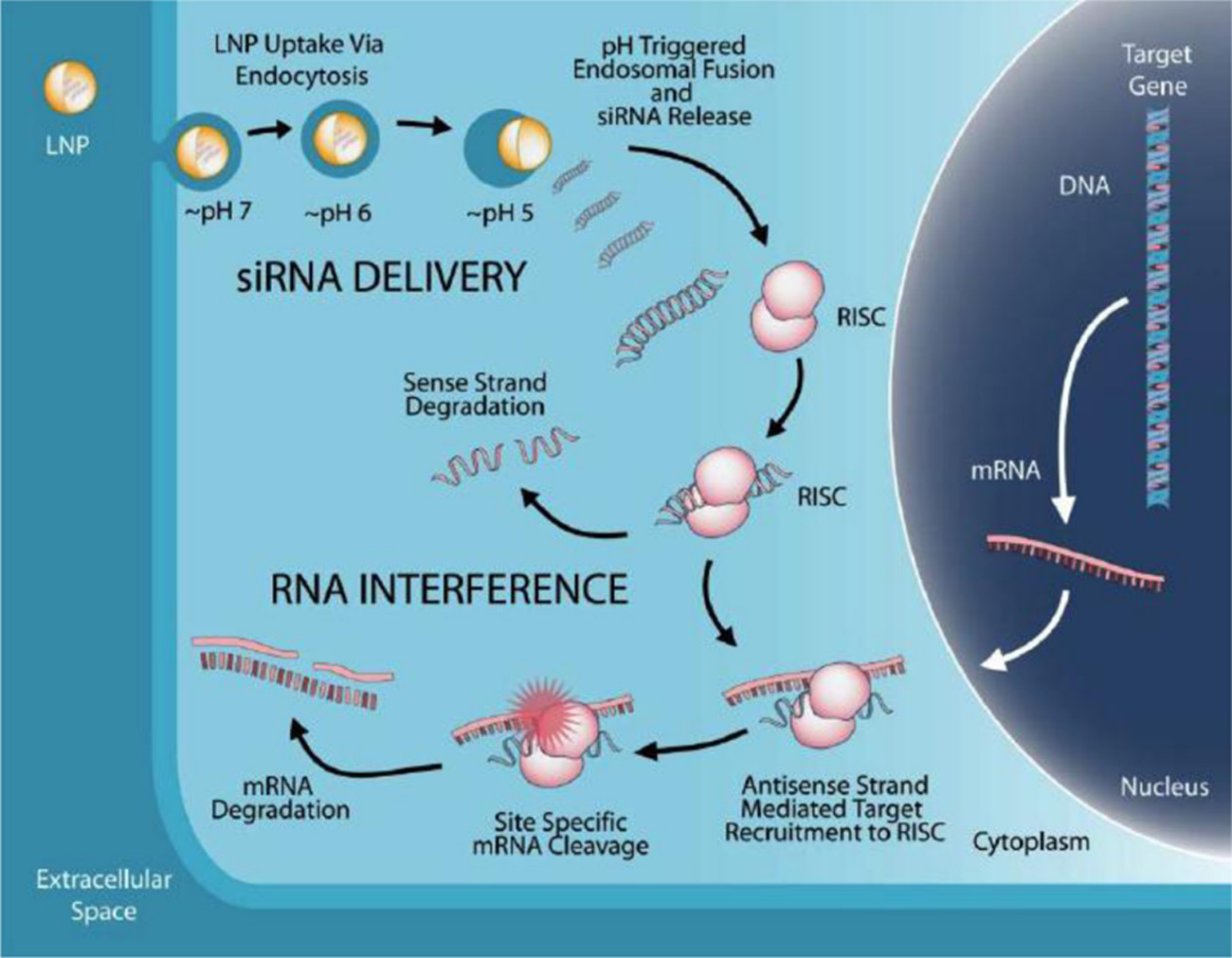
Mechanism of LNP-Mediated RNA Interface [22].

The efficacy of TKM-130803 for the treatment of Ebola virus disease (EVD) was trialled in a single arm phase II trial in laboratory confirmed EVD subjects in Sierra Leone during the 2014–16 Ebola-Zaire (Makona) outbreak [1,2]. TKM products are formulations of two small interfering RNAs (siRNA) encapsulated by four lipids to form nanoparticles of approximately 80 nm in diameter. The LNP formulation was developed for the intravenous (IV) delivery of the siRNA component into the cytoplasm of cells. TKM-130803 comprises of two siRNA molecules siLpol-2 and siVP35-2 in a 1:1 mixture by weight and molarity. The two siRNAs silence expression of EBOV mRNA-dependent L polymerase (Lpol), and Viral Protein 35 (VP35) respectively. The siRNAs enhance host mediated viral mRNA destruction thereby inhibiting Ebola virus (EBOV) replication (Fig. 1) [3], [4], [5]. This report describes the pharmacokinetics of siLpol-2 and siVP35-2 in EVD patients and explores the relationship between the pharmacokinetics (PK) of these molecules and survival. It also reports PK models for the siRNAs in EVD patients developed from the clinical trial data.

TKM-130803 was rapidly formulated at the beginning of the West African outbreak, to make a bespoke product with sequence alignment with the West African Ebola-Zaire (Makona), which caused the 2014–16 outbreak [1,6]. Its combined siRNA component was termed siEbola-3. TKM-130803 was developed from precursor investigational medicinal products (IMPs), TKM 100201 and TKM-100802 which were studied prior to the outbreak in post exposure prophylaxis models of Ebola-Zaire infections (EBOV-Zaire). TKM 100201 and TKM-100802 contained siRNA components with sequence alignments to the Kikwit strain of EBOV-Zaire (termed siEbola-2) [5,6]. The reformulation from siEbola-2 to siEbola-3 involved two nucleotide substitutions in the VP35 siRNA and one nucleotide substitution in the siLpol-2 [1], [3], [6]. Decisions regarding dose and regimen for the clinical trial were based on preclinical and healthy volunteer data for TKM-100201 and TKM-100802. Unfortunately the improved LNP2 which was used in conjunction with siEbola-3 to protect EBOV-infected non-human primates (NHPs) was not available for use in the clinical trial, which used LNP1 [6], [7].

The dose for the current study was conservative, chosen with reference to toxicology data from TKM 100201 and TKM-100802 to maximize safety. In a dose-escalation, healthy volunteer study, one (of two) participants dosed at the highest level of 0·5 mg/kg experienced cytokine release syndrome, so the maximal dose was limited to 0·3 mg/kg/day [3], [8]. Two patients who contracted EVD in West Africa were treated subsequently with TKM-100802 in the United States. The first patient was treated with doses starting at 0·3 mg/kg/day and rising to 0·5 mg/kg/day for 14 days. The second patient was treated with an unspecified dose for 6 days. No drug-related serious adverse events were reported in these patients [9]. NHPs were safely dosed at much higher levels (0·5–2·0 mg/kg/day) using the improved LNP2 formulation [5], [6], [7].

The European Medicines Agency recommended that a dose of 0·2 mg/kg/day may be effective in humans based on a series of experiments in NHPs and *in vitro* data [8]. The number of days post challenge was critical in determining the survival benefit of TKM-100802. Five out of six animals (83·3%) treated with TKM-100802 24 h post infection and three out of six treated 48 h post infection survived. When TKM-100802 was given 72 h after infection, four out of six (66·7%) animals survived. However, when TKM-100802 was given 96 h after infection, zero out of six animals survived [8]. siEbola-3 formulated with a LNP2 resulted in 100% survival (3/3) in rhesus monkeys infected with a lethal challenge of Makona variant EBOV. NHPs were dosed at 0·5 mg/kg/day for 7 days, commencing 72 h post-inoculation, a point in the disease course where viral RNA levels are typically 10^6^ RNA copies/ml [6].

The current PKPD study was embedded within a clinical trial of TKM-130803 carried out in EVD patients in Sierra Leone in 2015. 0·3 mg/kg of TKM-130803 was given daily. The trial was discontinued having reached a predefined statistical endpoint which indicated a low probability of demonstrating overall therapeutic benefit compared to historic controls [1]. This has to be interpreted in the context of the exceptionally high mean pre-treatment viral loads in the trial (>1 × 10^9^ RNA copies /ml plasma); over twice that seen in the trial of Zmapp monoclonal antibodies [7]. Preclinical data and healthy human studies have been used to set efficacy and toxicity thresholds. This is the first published study to our knowledge with sufficient PK data measured during an Ebola outbreak, to produce a PK *in silico* model.

## 2 Methods

### 2.1 Ethics statement and data sharing

The trial [1,2] was approved by the Sierra Leone Ethics and Scientific Review Committee, and the Oxford Tropical Research Ethics Committee. Approval to conduct the trial and import the trial drug was granted by the Pharmacy Board of Sierra Leone. The Committee for Medicinal Products for Human Use of the European Medicines Agency was asked for an opinion on the use of TKM-130803 in humans with EVD and was of the view that conducting a clinical trial of TKM-130803 in the context of the Ebola outbreak was acceptable. The UK Department for International Development and GOAL Global approved for the trial to be conducted at the Port Loko Ebola treatment centre (ETC). An independent data monitoring committee (IDMC) reviewed data on a sequential basis and reviewed any reported adverse events or other safety concerns. The trial was conducted in compliance with the International Conference on Harmonisation guidance on good clinical practice, and the Pharmacy Board of Sierra Leone conducted a good clinical practice compliance inspection during the trial. Written informed consent was obtained for all participants. The original data set is available on request.

#### 2.1.1 Trial design

Fourteen participants with EVD were recruited into the clinical trial to receive the 0·3 mg/kg of TKM-130803 by intravenous infusion over 2 h once daily for up to 7 days. Blood was collected pre- and post-administration of TKM-130803 on days 1, 3, 5, and 7, and plasma was separated by centrifugation and stored in aliquots, for both quantification of viral load and quantification of drug in the plasma. For drug quantification the samples Trizol LS was added to plasma before being frozen at −80 °C for later shipping and evaluation. Plasma was available for quantification of drug concentrations for eight subjects, with sufficient samples post dose to permit modelling in seven subjects. Further details of trial design are reported here [1].

#### 2.1.2 Quantification of viral loads

Viral RNA was extracted using the Qiagen® EZ1™ Virus Mini Kit in combination with the EZ1™ Advanced XL Automated Purification System, then quantified using the Trombley Ebola Diagnostic assay [10], as used by Public Health England for diagnostics in Ebola Treatment Centres. Positive, negative and extraction controls were included as standard for each quantification for quality control.

#### 2.1.3 Quantification of siRNA concentrations

To detect the concentration of siLpol-2 and siVP35-2, total RNA was extracted from the plasma using a partial Trizol ™ LS Reagent (Invitrogen) extraction method. Plasma was mixed with Trizol LS at the time of collection. At the time of quantification, samples were thawed and centrifuged, then the aqueous phase, containing the drug, was collected then hybridised to complementary oligonucleotides (siVP35-2 or siLpol Capture Probe and Detection Probe, Exiqon), first a biotinylated “capture” probe, then a digoxigenin-labelled “detection” probe. Hybridized samples were transferred to Neutravidin-coated, black-walled microtitre plate (Pierce), and incubated to allow capture of the complexes. Plates were washed, then anti-digoxigenin conjugated to alkaline phosphatase (Roche Biochemicals) added, followed by the addition of AttoPhos substrate (Promega) for a fluorometric readout. Plates were read using a BioTek® FLx800 reader and analysed using BioTek Gen5 Data Analysis software. The calibration range of the assay is 0·5–100 ng/mL, while the quantification range is 1–100 ng/mL. Samples outside this range were diluted as required in sample diluent (Trizol LS reagent, human K_2_EDTA plasma) before the repeating the extraction and hybridization process. Quality control samples containing 3, 50 and 80 ng/mL diluted in sample diluent were included in each assay. All QC and standards were expected to be within 20% of the theoretical values for the assay.

#### 2.1.4 Clinical data collection

Subjects were categorized according to survival at 14 days, which was the primary endpoint of the clinical trial [1]. Due to the restraints intrinsic in collecting timed blood draws from EVD subjects, the exact sampling times pre- and post-dose varied. The time of each blood draw was noted in the clinical trial record. Viral load at T0 was calculated by interpolating viral load measured in the pre-dose and post dose samples. ‘Time zero (T0)’ for each subject was defined as the time at which the initial dose of TKM-130803 was commenced. The area under the viral load curve was calculated using the trapezioidal method with Stata IC version 15 (Statacorp, Texas) for the period of treatment (7 days) after T0 or until death, whichever came first. Since the time of follow up necessarily varied, AUC/hour was used for comparison between subjects. Comparisons of subjects who survived to the 14-day endpoint ‘Survived’ and those who did not ‘Died’ was carried out using Mann-Whitney-U comparison of mean ranks using Stata IC version 15. For reference, 1 ng siPol-2 = 4·21 × 10^10^ Molecules, 1 ng suVP35-2 = 4·20 × 10^10^ molecules, thus a 1:1 ratio by weight equates to molar equivalent in the lipid nanoparticle.

Molecular excess was shown from calculation of the number of molecules of siRNA component compared to the number of molecules of virus. The number molecules of siRNA was calculated from mass per mL and molecular mass of the siRNA component and number of virus molecules was calculated as above.

#### 2.1.5 PK thresholds

Peak and mean concentrations of siRNA components were compared to the efficacy thresholds predicted from *in vitro* experiments using TKM-100802: EC_50_ (WT Kikwit 1995 strain): 0·04–0·57 ng/mL and EC_90_ (GFP-EBOV Mayinga variant strain) 1·43 ng/mL [11]. For the purpose of this exercise we assumed that efficacy is driven by AUC, and toxicity by Cmax. The dose and regimen that would maximise AUC, with a Cmax of 3000 ng/ml was considered. This threshold is the minimum 50% cytotoxic concentration (CC50) level for Hep2G cells, henceforth referred to as the “CC_50_ threshold” over a seven day treatment period [11].

#### 2.1.6 Pharmacokinetic model

Population pharmacokinetic modelling, calculation of area under the curve (AUC) values for each siRNA, and simulations, were performed using Pmetrics [12] within R version 3.1.0. Separate PK models were built for both siLpol-2 and siVP35-2 components of TKM-130803.

**Fig. 2:**
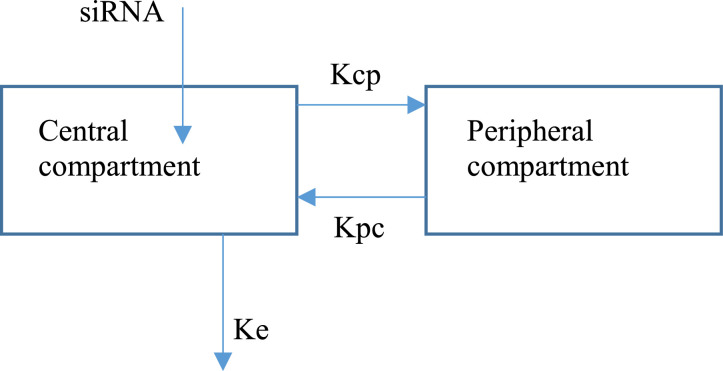
A representation of a two compartment model as used for both siRNA simulations. SiRNA is infused into the central compartment and out according to constant of elimination (*K_e_*). The first order rate constants of distribution from and to the central compartment denoted by *K_cp_* and *K_pc_,* respectively.

The final structural models for both siLpol-2 and siVP35-2 incorporated a two-compartment model with infusion of the TKM-130803 component in to the central compartment (Fig. 2) as detailed by the differential Eqs. (1a) and (1b):

(1a) $$\frac{dX_{1}}{dt}=k_{pc}X_{2}-\left(\frac{CL}{V}\right)X_{1}-k_{cp}X_{1}$$

(1b) $$\frac{dX_{2}}{dt}=k_{cp}X_{1}-k_{pc}X_{2}$$

where *X*_1_ and *X*_2_ are the amounts of siLpol-2 or siVP35-2 respectively in the central and peripheral compartments, representing well perfused organs/systemic circulation and less well perfused tissues/organs, respectively. The pharmacokinetic parameters CL and V denote the clearance and volume of distribution, respectively. *k_cp_* and *k_pc_* represent the first order rate constants of distribution from and to the central compartment.

**Fig. 3:**
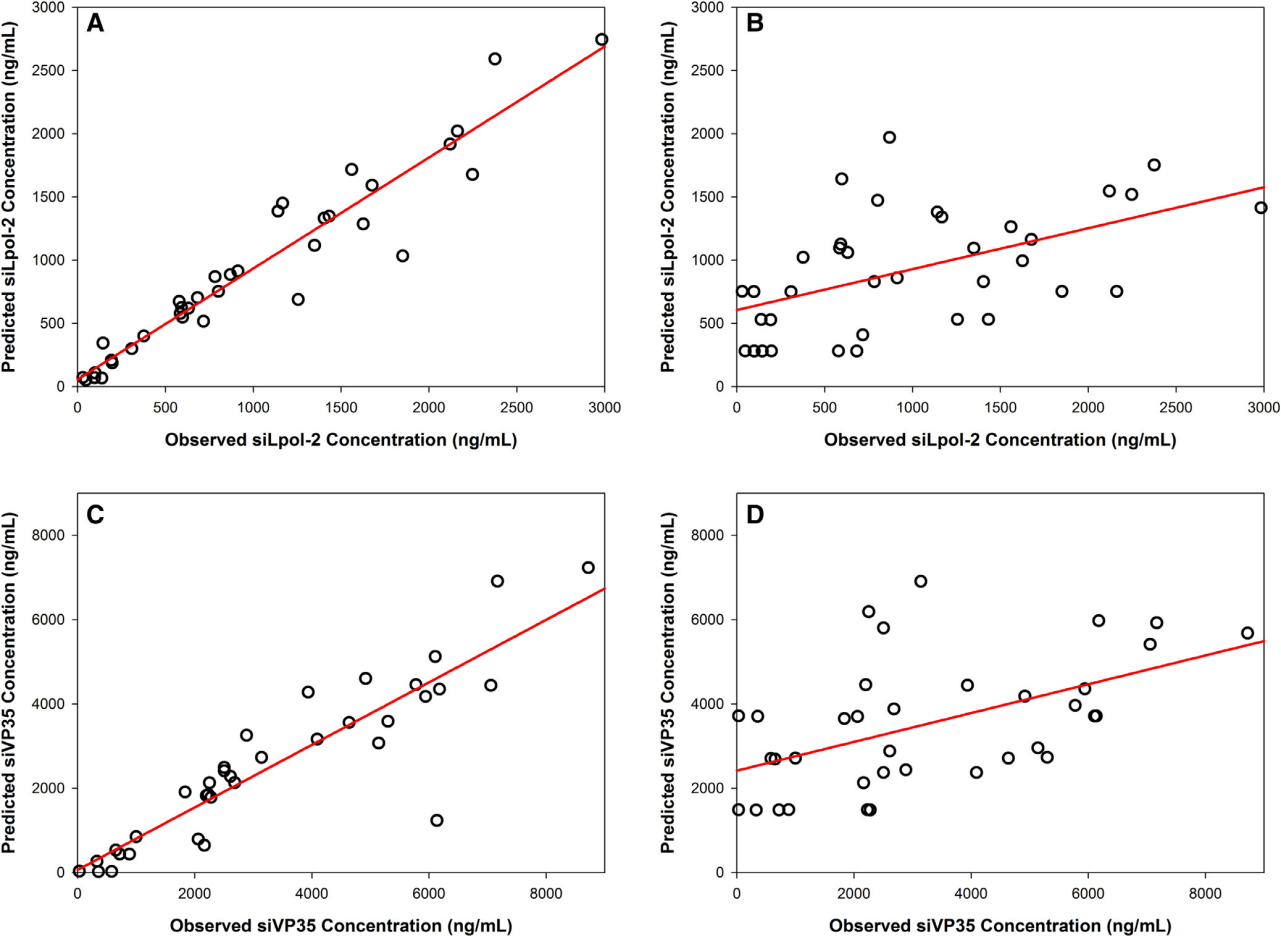
Predicted *versus* observed concentrations for individual and population prediction for siLpol-2: (A) and (B) and siVP35: (C) and (D). *R*^2^ values for siLpol-2 were determined to be 0·90 and 0·53 for individual (A) and population (B) predictions. For siVP35 *R*^2^ was determined to be 0·91 and 0·52 for individual (C) and population (D) predictions.

Model fitting was performed using protocols defined previously [13,14]. Briefly, the goodness-of-fit of the observed/predicted values (population and individual predictions) were assessed by linear regression (intercept close to 0, slope close to 1), the coefficient of determination of the linear regression (R^2^ close to 1·0) and log-likelihood values; plots for final models are shown in Fig. 3. The ratio achieved of siRNA molecules: genomes of viral load was calculated compared in the Survived and Died subjects.

Monte Carlo simulation of 1000 individuals using the PK models for siLpoL-2 and VP35, was performed to simulate a) the standard dose (0·3 mg/kg/day) in a 2 h infusion for purposes of a visual predictive check (VPC) and at 0·5 and 1 times the standard dose in a continuous infusion regimen designed to maximise drug exposure, whilst minimising peak drug concentrations. PK profiles were compared to efficacy and toxicology thresholds (supplementary information). We extrapolated a pharmacokinetic target from previous data. We considered which dose and regimen would maximise the AUC, with a Cmax of 3000 ng/ml (minimum CC50 DLT for Hep2G cells) and minimise the peak concentration using an infused dose over a seven-day treatment period.

## 3 Results

### 3.1 Observed PK and PD parameters

Viral load (VL) was measured at a median of 1·18 h before T0 (when treatment was commenced) (IQR: 3·4 to 0·8 h) and again a median of 2·22 h after T0 (IQR 2·07 to 2·65). The PCR cycle threshold (cT) value was log-linearly related to VL. Log_10_ VL ranged from 8·04 to 9·49 (median 8·79, IQR 8·42 to 9·36).

**Fig. 4:**
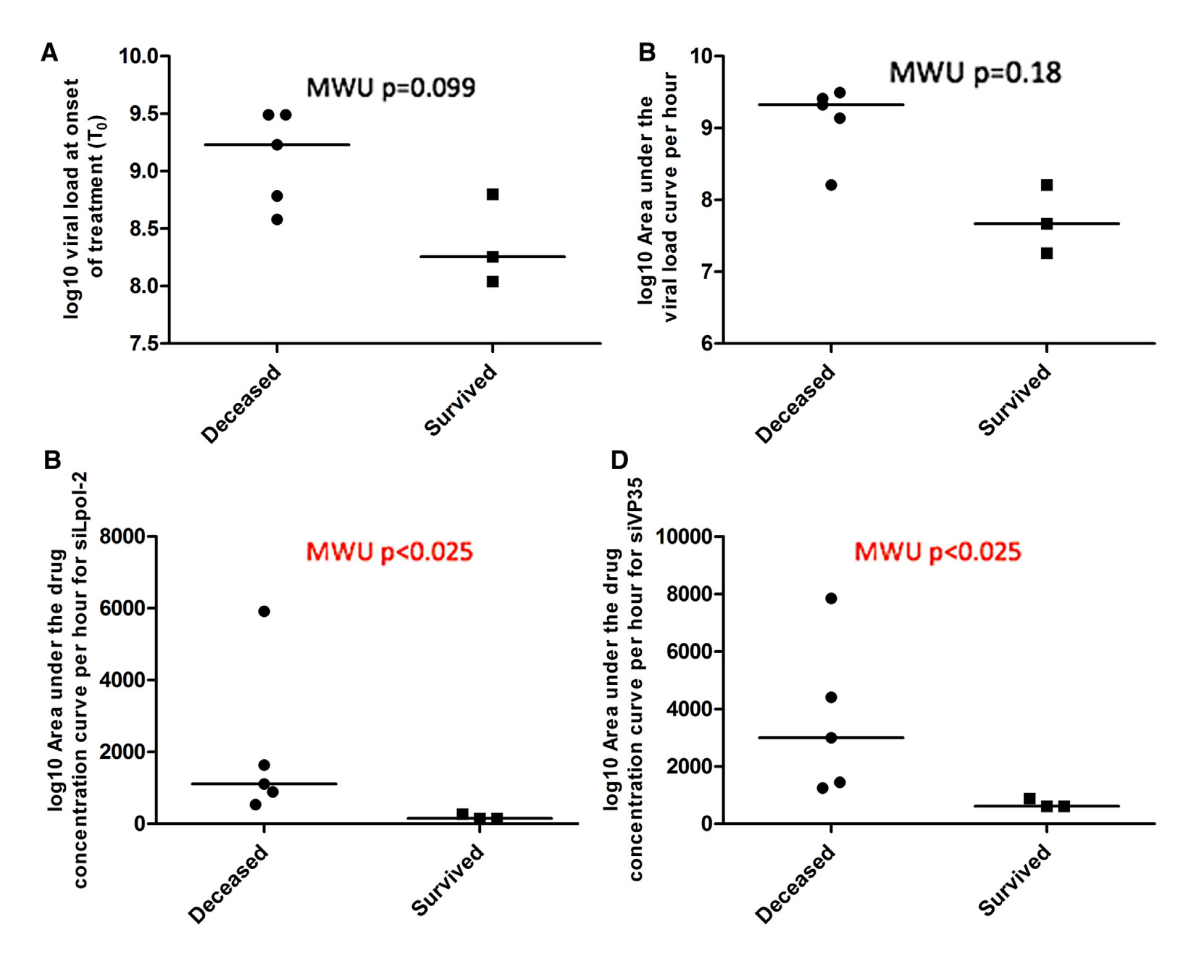
A comparison of viral load and component siRNA strands of TKM-130803 compared by outcome (survived or died) A: log_10_VL at T0 B: log_10_ (AUC of VL) C: AUC/hr siLpol-2 D: AUC/hr siVP35-2. Median values depicted by a horizontal line.

Viral load at T0, was lower in subjects who survived compared to those who died but this difference was not statistically significant (*p* = 0·099) (Fig. 4(A)). The viral load remained lower in those who survived: the AUC of VL per hour was lower in subjects who survived compared to those who died, again this difference was not statistically significant (*p* = 0·10) (Fig. 4(B)). The concentration of both siRNAs was significantly higher in subjects who died compared to those who survived, probably indicating poorer drug clearance in their advanced disease state. The AUC of siLpol-2 and siVP35-2 per hour was significantly higher in subjects who died compared to subjects who survived (*p*<0·025 for both siRNA) (Fig. 4(C) and (D)). The associated median levels for Fig. 4 and IQR are in Supplementary Material Table 1. This is also observed in considering the relationship between change in viral load from initial dose to last recorded viral load and viral AUC per hour.

**Fig. 5:**
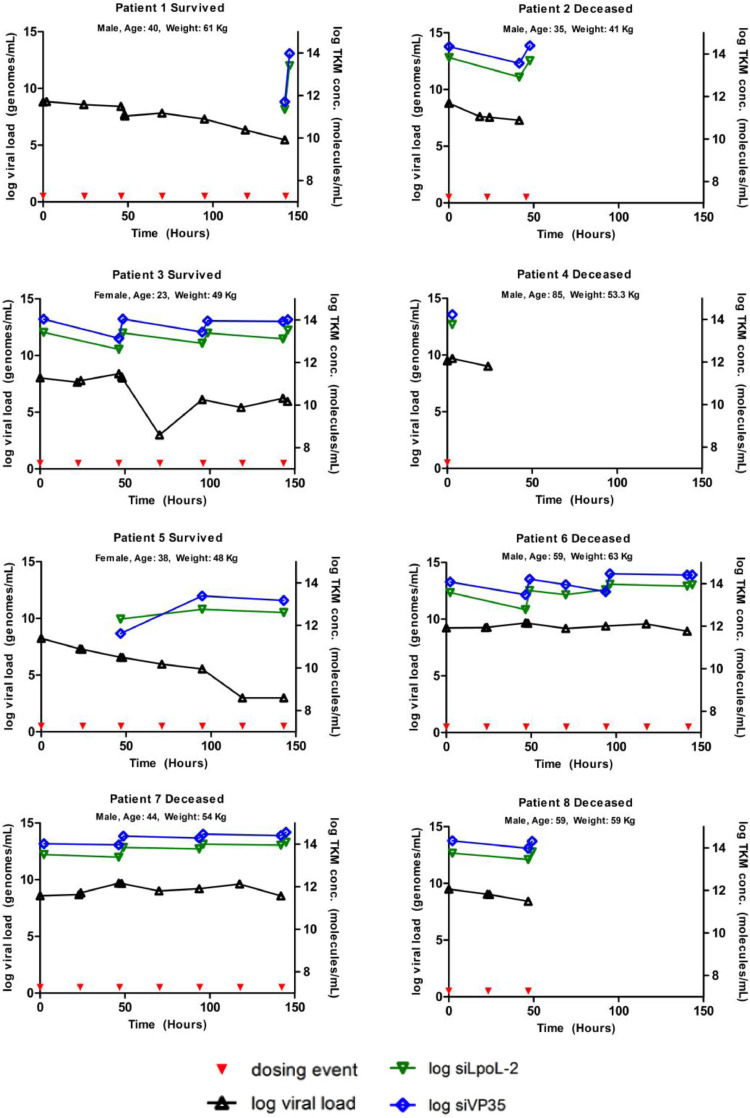
siPol-2 & siVP35-2 (Molecules/ml), and Viral Load (VL) (genomes/ml) profiles over time per subject.

There was a molar excess of both siRNAs over the course of treatment in the plasma (Fig. 5), however the ratio of siRNA molecules/genomes was not significantly different between those who died and those who survived (siLpol-2: *p* = 0·88; siVP35-2: *p* = 0·65). Two subjects had markedly higher ratios of molecule/genome (subjects #2, who died and #3 who survived). The highest ratio of molecule/genome was 221,677.8 (siPol-2, in subject #2) and 1,183,506 (VP35-2 in subject #3).

### 3.2 The PK model

Two compartment models were fitted to siLpol-2 and siVP35 concentrations using population pharmacokinetic analysis. There were excellent correlations between observed and predicted values for these models (Fig. 3). PK parameters for two compartment models for silpol-2 and siVP35 are shown in Supplementary Material Table 1, clearance (CL) and volume of distribution (*V_d_*) are substantially higher for siLpol-2 than for siVP35 (12·43 ml/h/kg and 56·69 ml/kg and compared to 3·39 mL/h/kg and 16·69 mL/h). This indicates that siVP35 will display greater drug accumulation in EVD patients than siLpol-2. Elimination rate constants were determined to be similar for the two siRNA components, as were rate constants to and from the peripheral compartment.

### 3.3 *In silico* simulated PK in the context of preclinical data

**Fig. 6:**
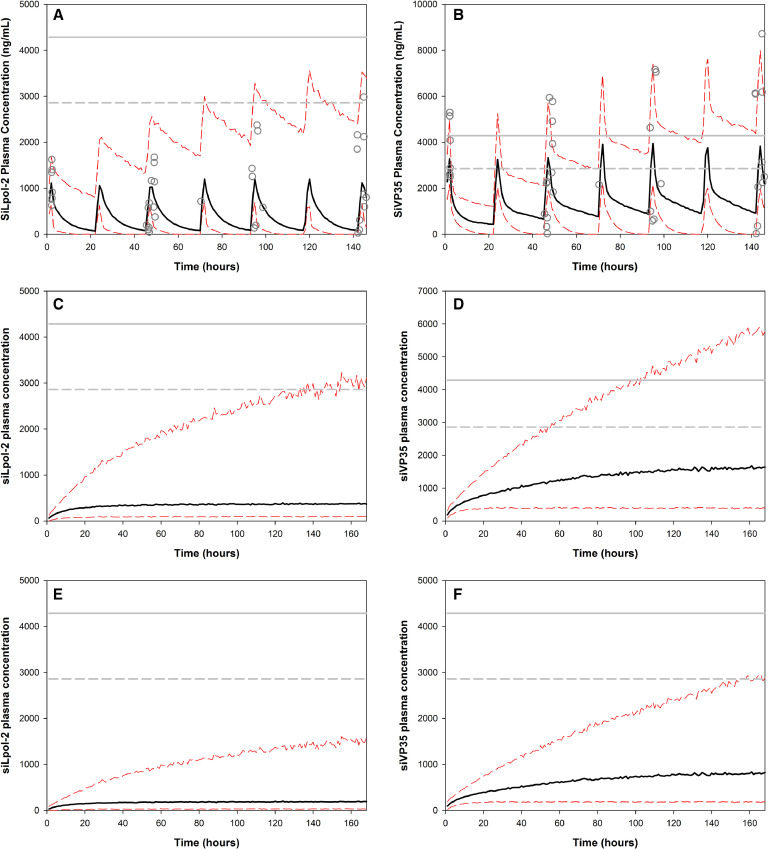
Simulated pharmacokinetic profiles based on 1000 simulated individuals for different TKM regimens from population models based on clinical pharmacokinetic data. Simulated concentration profiles of (A) siLpol-2 and (B) siVP35 for once daily 2 h infusion of 0·3 mg/Kg/day total TKM (0·15 mg/Kg/day siLpol-2 or siVP35). Simulated concentration profile of (C) siLpol-2 and (D) siVP35 for continuous infusion regimen of 0·3 mg/kg/day total TKM (0·15 mg/kg/day siLpol-2/siVP35). Simulated concentration profile of (E) siLpol-2 and F) siVP35 for continuous infusion regimen of 0·15 mg/Kg/day total TKM (0·075 mg/kg/day siLpol-2/siVP35). Black solid lines represent median simulated drug concentration profiles and red dashed lines represent 5th and 95th percentile concentration profiles, respectively. Grey circles in profiles A and B represent observed drug concentrations from sparse plasma sampling. Horizontal solid and dashed grey lines represent the upper and lower limits of the cytotoxic concentration (CC_50_) as determined in Hep2G cells.

Simulations of the PK of TKM-130803 in patients in the current trial exceed the *in vitro* efficacy thresholds of 0·04–0·57 ng/mL and 1·43 ng/mL (EC_50_ and EC_90_) [11] by several orders of magnitude in all patients in the current clinical trial at the standard dose (0·3 mg/kg/day) (Fig. 6(A) and (B)).

#### 3.3.1 Median concentrations at a dose of 0·3 mg/kg/day infused once a day over 2 h

The median peak concentrations achieved by siLpoL-2 (Fig. 6(A)) and siVP35 (Fig. 6(B)) in patients using a 0·3 mg/kg/day TKM-130803 dosage regimen were lower than the CC_50_ threshold. Dosing in the current trial reproduced Cmax values consistent with those predicted in the NHP models using TKM-100802 for siLpol-2, with the median peak concentration being in line with Cmax values from NOAEL for repeat dosing (Fig. 6(A)). The simulated median peak concentrations (Cmax) were: 1471 ng/mL (IQR: 756 ng/mL) for siLpol-2 and 4585 ng/mL (IQR: 2936 ng/mL) for siVP35. There was more accumulation of siVP35 than siLpol-2. The peak concentration of siVP35 was slightly above the repeat dose NOAEL Cmax level observed in NHPs, using the 0·3 mg/kg/day TKM-130803 dosage regimen.

#### 3.3.2 percentile concentrations at a dose of 0·3 mg/kg/day infused once a day over 2 h

Drug accumulated more in patients who died than those who survived (Fig. 4(C) and (D)). This is likely to have driven the accumulation over time of drug seen reflected in the 95th percentile of the overall PK simulation (Fig. 6(A) and (B)). The 95th percentile exceeded the CC_50_ threshold for both siPol-2 and VP35. Despite this, no adverse events were observed during the clinical trial [1].

#### 3.3.3 Simulating an increase in infusion time

AUC can be maintained with lowering in peak drug concentration using longer infusion times. Taking this to its extreme for illustration we considered a continuous infusion for 7 days instead of a 2 h infusion once a day for 7 days. For siLpol-2 the AUC remained the same for the daily 2-h infusion and the continuous infusion for 7 days from 60,609 ng*h/mL (IQR: 97,606 ng*h/mL) (2 h infusion) to 58,515 ng*h/mL (IQR: 94,606 ng*h/mL), (continuous infusion) over the 7 day period. The simulated peak concentrations for the two regimens are however markedly different: median values 1471 ng/mL (IQR: 757 ng/mL) for the standard regimen and 603 ng/mL (IQR: 932 ng/mL) for continuous infusion. For siVP35 the AUC for the daily 2-hour infusion and the continuous infusion for 7 days are: 239,690 ng*h/mL (IQR: 265,040 ng*h/mL)(2 h infusion), and 233,743 ng*h/mL (IQR: 253,205 ng*h/mL) (continuous infusion), respectively. The simulated peak concentrations for the two regimens are again markedly different, with median values 4585 ng/mL (IQR: 2936 ng/mL) for the standard regimen and 2373 ng/mL (IQR: 3023 ng/mL) for the continuous infusion (Fig. 6).

The maximum possible dose for which the 95% percentile remained under the CC_50_ threshold was an infusion of 0·15 mg/Kg/day (Fig. 6(E) and (F)). This resulted in an AUC of 29,141 ng*hr/mL (IQR: 47,374) for siPol-2 and 116,812 (IQR: 126,441) for VP35, with Cmax median values of 342 ng/mL (IQR: 466 ng/mL) and 1223 ng/mL (IQR: 1507 ng/mL) (Fig. 6).

## 4 Discussion

TKM was tested in preclinical trials in NHPs within hours of being inoculated with Ebola virus with good effect [5]. Whilst it is disappointing that a drug which looked so promising in NHPs would not prove efficacious in a human trial [1], the preclinical NHP data predicted success in a challenge of up to 10^6^ RNA copies/ml EBOV with TKM dosed at 0·5 mg/kg/day within 72 h of infection [15] It is important that when TKM-100802 was given 96 h after infection, 0/6 animals survived [8]. EVD patients often present to Ebola treatment centres several days after onset of symptoms, between 2 and 21 days post infection [16]. Pre-treatment viral loads in the clinical trial were above 10^9^ RNA copies/ml^1^ (over twice that reported in the ZMapp study [7]). Patients treated with TKM-130803 in Sierra Leone, may have presented too late for an antiviral to have an effect. The viral loads tested in the human trial far exceeded that tested in animal models and at which time, end-stage organ damage was irreversible [1,17]. Supporting this hypothesis: Viral loads of >8 log_10_ copies ml^−1^ were ≥90% predictive of a fatal outcome in the 2000–2001 SUDV Gulu outbreak [18]. However the serial sampling of PKPD samples has permitted an insight into pharmacokinetics in the disease state which could be useful for future trials of siRNAs or other anti-Ebola therapeutics [1,7].

PK has been deployed with utility for other EVD therapeutics, most notably Favipiravir which compared drug concentrations to a model developed from healthy human volunteers [19]. However the current study is the only EVD therapeutic to date with both PK and PD information from human subjects with acute EVD, from which an *in silico* PK model has been published. The severe haemodynamic distruption caused by EVD, culminating in multi-organ failure [20] is likely to alter the PK of any drug administered to a patient, compared to the PK in a healthy subject.

In this study, the number of molecules of either siRNA (siLpol-2 and siVP35-2) of TKM-130803 in plasma exceeded the number of Ebola virus genomes per ml throughout treatment in all subjects assessed. We propose that this suggests there was sufficient siRNA to be effective. However, this may not reflect the ratio at the site of action, which is intracellular. The highest drug/viral load ratio achieved was 1 × 10^6^ molecules/genome. Prior to treatment and throughout disease, there was no statistically significant difference in viral load between subjects who subsequently survived or died, although there was a non-significant trend for those who died to have higher viral loads. This is consistent with the small number of patients in the same treatment centre who were not treated [1] and with observations in the wider epidemic [21].

Both siRNA concentrations were significantly higher over the course of treatment in subjects who died. Explanations for this include: (1) patients who died had impaired drug clearance, or, since the siRNA function intracellularly, (2) cellular uptake was lower resulting in higher concentrations of circulating drug. The LPD capsule was designed to protect the siRNAs rapid renal clearance to enable effective intracellular uptake [3,22], and failing organs, including renal failure are predictive of mortality [20,23], therefore either impared renal clearance or cellular uptake are feasible hypothesis for higher concentrations of circulating drug. A third possibility is that those who died suffered more drug related toxicity. However given that no drug related serious adverse reactions were detected, including cytokine release syndrome, and viral loads were high at admission and remained high over time in patients who died, it is likely that the association between high TKM-130803 concentrations and death is a reflection of impaired drug clearance in sicker patients or impaired cellular uptake, rather than drug related toxicity [24]. Higher plasma drug concentrations, by either mechanism, may put participants with more advanced disease at a greater risk of a serious adverse event.

The trial used a dose of TKM-130803 that was determined to balance safety and potential clinical benefit. Cytokine release syndrome was observed as an adverse event in one healthy volunteer from an earlier Phase 1 trial of TKM-100802 [3], [7], which resulted in the recommended dose being reduced from 0·5 mg/kg/d to 0·3 mg//kg/day. Cytokine release syndrome was not observed in any participant in this trial of TKM-130803 at a dose of 0·3 mg/kg/day [1]. Dose selection was also informed, in part, by the need for a single daily, short infusion, due to the logistical difficulties in supervising an intravenous infusion of an experimental drug in an Ebola Treatment Unit [2] and the NOAEL observed in NHPs. The high mortality and morbidity associated with EVD could have justified using a higher dose, but with a greater risk of adverse events [4,25]. As a thought experiment, PK curves were simulated as continuous infusions; an idealised situation in which the AUC can be maintained whilst minimising peak drug concentrations. Although impractical in the real world, this served to indicate that using the PK observed in these patients, a higher dose could not have been used without breaching the toxicology threshold. The PKPD data from participants in this trial indicate that plasma concentrations of TKM-130803 were in substantial molar excess of viral RNA, and clearance was impaired, suggesting the conservative approach was justified.

It remains possible that TKM-130803 could be efficacious if used as a post-exposure prophylaxis or is commenced earlier in the disease process, in which case it is likely that a higher dose could be tolerated in patients with intact drug clearance. We suggest that future drug trials for siRNA, or other therapeutics stratify analysis by pre-treatment viral load. The safety of dosing high viral load patients with advanced disease should be considered assuming reduced drug clearance, and a commensuratly higher propensity to adverse effects. siRNA-type therapeutics have the advantage that they can be swiftly developed and produced in response to previously unknown viruses or strains of viruses [15]. Given their novel targets, they could be used synergistically with other drug types. The lipid nanoparticle (LNP) technique in particular continues to be improved: siRNAs are protected from degradation by plasma and tissue nucleases and facilitate intracellular uptake of the nanoparticle by endocytosis preventing the rapid clearance of the siRNAs [22]. A recent reformulation, using a VP35-targeting siRNA with a new lipid nanoparticle component, has achieved 100% survival of NHPs challenged with Ebola-Sudan, even when the animals were dosed 5 days after infection, although animals with viral loads greater than 8·9 log_10_ GEq ml^−1^ succumbed [15].

That this drug was not efficacious in human subjects with severe EVD using a conservative dosing regimen does not preclude the possibility that an alternative dose, given earlier, a different regimen, or using an improved lipid nanoparticle formulation might have some efficacy. It seems likely that the patients in the clinical trial with extremely high viral loads, died because they were physiologically beyond the point of no return. We propose that efficacy of TKM 130803 was neither proved nor disproved by the clinical trial, but that it has given a useful insight into the pharmacokinetics of the siRNA in the disease state [1].

## Appendix Supplementary materials

Image, application 1
