# Supplementary material for: Pharmacokinetics of TKM-130803 in Sierra Leonean patients with Ebola virus disease: plasma concentrations exceed target levels, with drug accumulation in the most severe patients
Source: eBioMedicine. 2020 Jan 14;52:102601. doi: 10.1016/j.ebiom.2019.102601 (PMC7033524; doi:10.1016/j.ebiom.2019.102601)
Supplement: Supplementary file 1 [file mmc1.docx]

**Supplementary Material.**

**Supplementary Table 1:** A comparison of viral load and component siRNA strands of TKM-130803 compared by outcome (survived or died).

| **Parameter** | **Survived (N=3)**  **Median (IQR)** | **Died (N=5)** | **p** | **Figure** |
| --- | --- | --- | --- | --- |
| log_10_ Viral Load (VL) pretreatment (T0)  (genomes/ml) | log_10_VL 8·26  (IQR IQR 8·04-8·80) | log_10_VL 9·23  (IQR 8·79-9·49) | 0·099 | 4A |
| log_10_ (AUC of VL)  (genomes/ml/hr | 7·67  (IQR 7·26-8·21) | 9·33  (IQR 9·14- 9·41) | 0·18 | 4B |
| AUC siLpol-2  (ng/ml/hr) | 150·13  (IQR 149·74-272·48) | 1,112·24  IQR 1,636·85- 5,918·14 | 0·025 | 4C |
| AUC siVP35-2  (ng/ml/hr) | 2 613·61  (IQR 611·87-880·99) | 2 2,999·94  IQR 1,442·28- 440·16 | 0·025 | 4D |
| Molecules of siLpol-2/ genome EBOV | 37,340  **(**IQR 2,943 – 221,678) | 36,907  IQR 31,479-46,531 | 0·88 | 5 |
| Molecules of siVP35-2 genome EBOV | 145,570  (IQR 9,682–1,183,506) | 112,696  (IQR 109,736-119,151) | 0·65 | 5 |

**Supplementary Material – Table 2:** Population pharmacokinetic parameters for TKM component siRNAs from two compartment population pharmacokinetic models fitted to EBOV patient drug concentrations. CL, Vc denote clearance and volume distribution. K_e_, k_cp_and k_pc_ denote first order rate constants for elimination from the central compartment and transfer to and from the peripheral compartment, respectively. Parameter mean values given (standard deviations show in parentheses), [95% CI values in square parentheses].

CI values were generated via a Monte Carlo simulation to create 1000 x npoint samples with replacement from the weighted marginal distribution of each parameter, where npoint is the number of support points in the model. The simulations performed did not account for this uncertainty in parameter estimates.

| **Parameter** | **siLpol-2** | **siVP35** |
| --- | --- | --- |
| CL (mL/hr/Kg) | 12·43 (16·77) | 3·39 (4·92) |
| K_e_ | 0·28 (0·41) [0·00001─0·98] | 0·21 (0·29) [0·017─0·684] |
| V_c_ (mL/Kg) | 56·69 (20·48) [39·46-81·22] | 16·69 (1·55) [15·65─16·62] |
| k_cp_ | 1·29 (0·29) [0·98─1·61] | 1·29 (0·42) [0·82─1·90] |
| k_pc_ | 0·94 (1·63) [0·05─2·12] | 0·39 (0·40) [0·04─1·00] |

**Supplementary Material – Figure 1.**

Individual fits for A) siLpol-2 siRNA and B) VP35 component concentrations. Observed concentrations shown in black open circles and model predictions show as a solid black line.


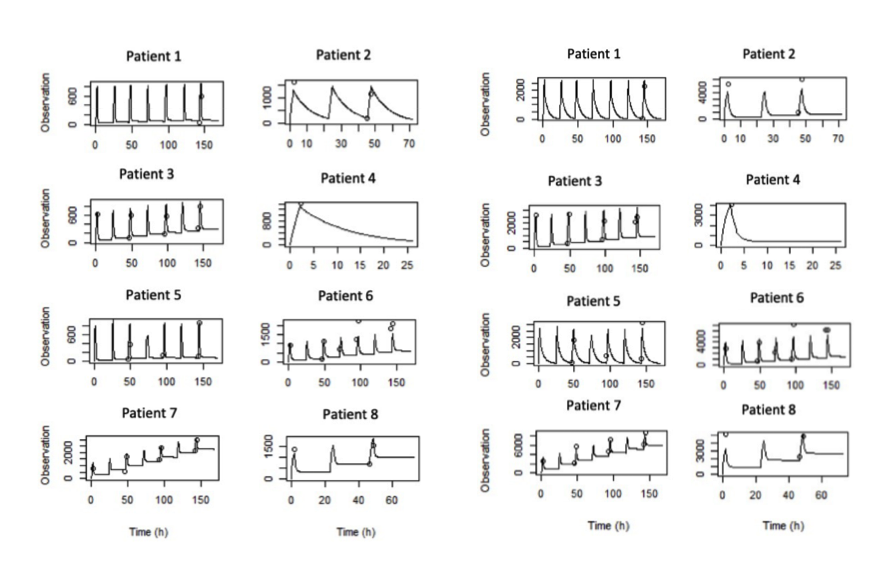


B.

A.

**Supplementary Material – Figure 2.**

Visual predictive check plots depicting concentration against time after first dose, for a) siLpol-2 and b) siVP35. Observed concentrations shown as blue circles and simulated 5^th^, 50^th^ and 95^th^ percentile concentration levels are shown in dark grey. Confidence intervals for simulated percent are shown in light grey.

a)


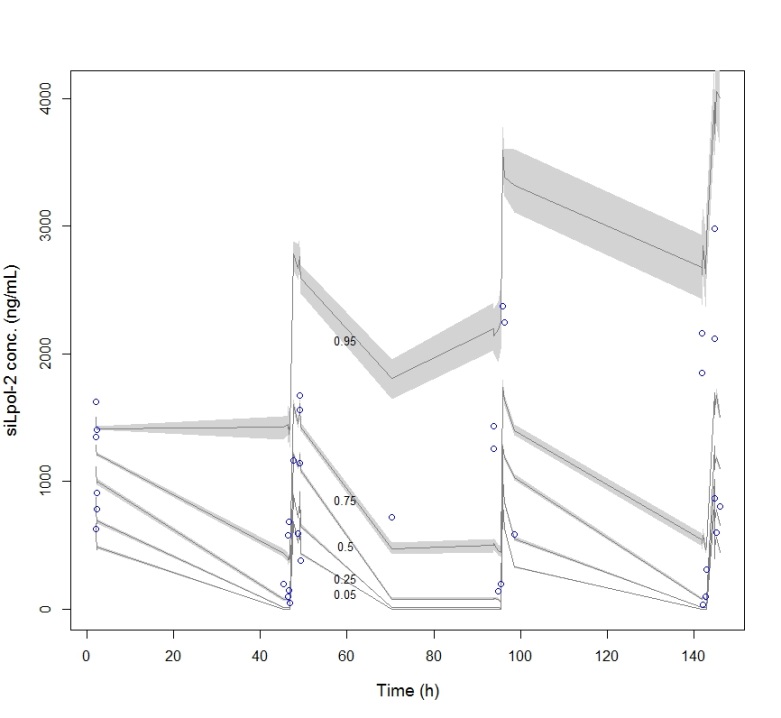


b)


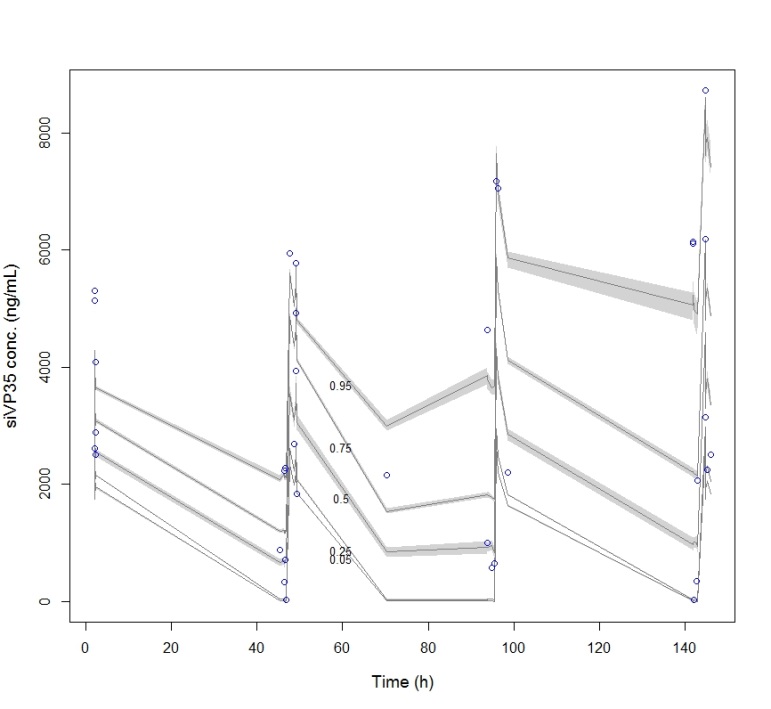


**Supplementary Material – Table 3:** Justification of the choice of 2 compartment models over 1 compartment models. -2LL, AIC and BIC values for 1 and 2 compartment population models for siLpol-2 and siVP35. There is “strong evidence” (ΔBIC > 6-10) for the 2-compartment models in the siLpol-2 model and “strong to positive” evidence for the siVP35 model (ΔBIC > 2-6). In practice, a drop in AIC or BIC of 2 is an accepted a threshold for considering one model over another.

| **Parameter** | **siLpol-2** | | **siVP35** | |
| --- | --- | --- | --- | --- |
|  | **1 compartment** | **2 compartment** | **1 compartment** | **2 compartment** |
| -2LL | 537·8299 | 492·144 | 598·5058 | 585·7327 |
| AIC | 544·5572 | 504·144 | 605·2558 | 597·7327 |
| BIC | 548·6627 | 510·0616 | 609·2563 | 603·6503 |
